# Quality improvement by introduction of 72-hour admission pathway in Forest House Adolescent Unit (FHAU) for young people in crisis

**DOI:** 10.1192/bjo.2021.566

**Published:** 2021-06-18

**Authors:** Sadaf Mufti, Linda Zirinsky

**Affiliations:** HPFT

## Abstract

**Aims:**

Short admissions for crisis management among young people suffering with Emotionally Unstable Personality Disorder (EUPD) as recommended in National Institute for Health and Care Excellence (NICE) 2009 guidelines are not routinely offered in the United Kingdom (UK). Our aim was to introduce crisis admissions lasting for 72 hours. During this brief admission the families of young people presenting with suicidal behaviour are offered an assessment and diagnosis of young person's difficulties, psychoeducation, and safety plan for future risky behaviour, in addition to respite.

**Background:**

Three-day Crisis admission was set up with the aim of reducing inappropriate long admissions in people who may have more negative effects from admission than positive ones. A need was felt for a brief admission pathway in order to be able to provide treatment for patients suffering from EUPD traits in keeping with NICE guidelines. NICE guidelines suggest that people with borderline personality disorder should be considered for acute psychiatric inpatient admission only for the management of crises involving significant risk to self or others that cannot be managed by other services. The guidelines also recommend ensuring that the decision is based on an explicit and joint understanding of the potential benefits and likely harm that may result from admission and agreeing to the length and purpose of the admission in advance.

**Method:**

A retrospective study comparing length of hospital stay in the 2018 (when this model was introduced) with previous years, the number of serious incidents was carried out to assess the impact of this new admission model. The rate of readmissions in the same year was also assessed. For qualitative feedback regarding the effectiveness of the crisis admission as an intervention, a survey was carried out to assess parent satisfaction and the nursing staff was asked for their views.

**Result:**

There was a marked reduction in the number of serious incidents linked to suicide and length of hospital stay was reduced to half in the year when the crisis admissions were introduced as compared to the previous year. Only about 10-15% of patients required re-admission in the same year. About 90% of parents gave a positive feedback confirming the effectiveness of this intervention.

**Conclusion:**

72-hour crisis admissions for adolescents are effective, appropriate, clinically indicated alternative to routine admissions with a high parent satisfaction.

